# Extremity preservation in traumatic and nontraumatic lower extremity defects

**DOI:** 10.1007/s00508-025-02585-9

**Published:** 2025-08-18

**Authors:** Anna Fast, Eva Placheta-Györi, Thomas Rath, Christine Radtke

**Affiliations:** Department of Plastic, Reconstructive and Aesthetic Surgery, Medical University of Vienna, Vienna General Hospital, Währinger Gürtel 18-20, 1090 Vienna, Austria

**Keywords:** Limb salvage, Reconstructive surgery, Free tissue transfer, Microsurgery

## Abstract

**Background:**

Indications for reconstruction of the lower extremity range from posttraumatic defects to infections and tumors. Despite advancements in plastic surgery, flap surgery still poses a challenge. In this retrospective study local flap surgeries and microsurgical free flaps were assessed. Postoperative complications and limb preservation were analyzed.

**Methods:**

This retrospective study included 187 patients who were treated at a university-affiliated tertiary care hospital. Defects were of traumatic (29.4%) and nontraumatic (70.6%) etiology. Limb preservation was determined during a 12-month follow-up period. Patient characteristics, flap selection and postoperative flap-associated complications were collected.

**Results:**

The patient population included 107 men (57.2%) and 80 women (42.8%), 104 (55.6%) free flaps and 83 (44.4%) local flaps were performed. In the free flap group latissimus dorsi and gracilis flaps were most commonly performed. The most common surgeries in the local flap group were gastrocnemius, soleus and plantaris medialis muscle flaps. The overall limb preservation rate was 92.5% with no significant difference between the two groups.

**Conclusion:**

Both methods enable reconstruction of complex lower extremity wounds and enable limb preservation in many cases. The type of flap is selected based on the anatomical location of the defect, defect size and patient factors.

## Introduction

Plastic surgery plays a pivotal role in the field of lower limb reconstruction, encompassing a wide array of techniques aimed at restoring function, esthetics and quality of life for patients with various lower limb deformities and injuries [1, 2].

The feet play a crucial role in supporting the body’s weight and are essential for basic activities like walking, standing and running. Individuals with soft tissue deficiencies encounter difficulties in their daily tasks, while extremity amputations significantly impair function [3]. Reconstructive surgeons face unique challenges when dealing with tissue defects in the lower extremities. Success in reconstructive procedures hinges on a thorough understanding of anatomy, including muscle function and positioning in relation to surrounding structures. Planning for defect coverage relies on considerations, such as muscle size, vascular supply and skin area [4].

When discussing potential causes for lower extremity reconstruction, several factors come into play. Local issues such as pressure, infections and radiotherapy are common culprits. Metabolic factors, such as malnutrition and diabetes mellitus can also contribute [5]. Vascular problems, including peripheral artery occlusive disease, venous insufficiency and ulcers, may necessitate reconstruction. Dermatological conditions, such as squamous cell carcinoma, basal cell carcinoma and pyoderma gangrenosum can also require intervention. Iatrogenic causes, such as wounds resulting from steroid therapy or cytostatic therapy, are another consideration. Systemic factors, such as malignant processes, immunosuppression and hemodynamic disturbances may also play a role. Additionally, nicotine abuse, alcoholism, obesity, immobility, advanced age and trauma are potential contributing factors [6, 7].

### Local flaps

**Fig. 1:**
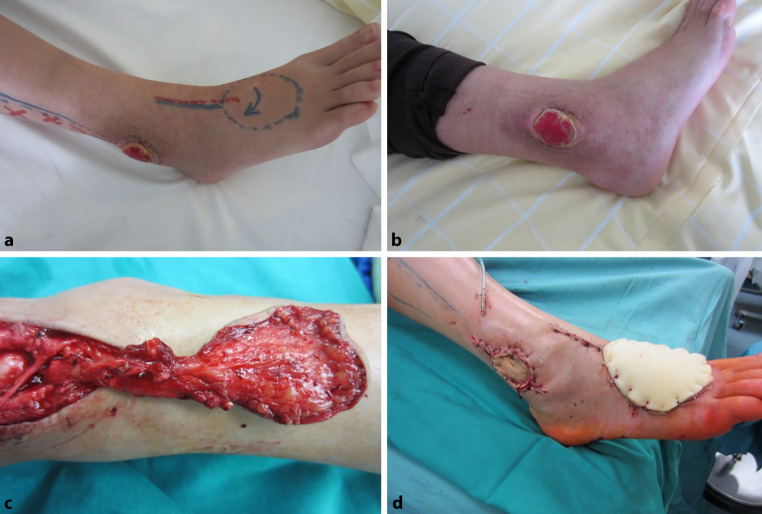
**a** Preoperative markings of the dorsalis pedis flap, **b** preoperative picture of the ulcer, **c** intraoperative flap plasty and **d** final operation situs

Understanding the vascular anatomy of flaps enables the relocation of local flaps to the required site, enabling the creation of the desired form and function [8]. For local flaps, lower extremity defect reconstruction should replace similar tissues and minimize donor site complications. Achieving a satisfactory esthetic appearance and functional outcome is important [3]. Local flap reconstructions, when applicable and appropriate for the size of the defect, have been linked to fewer reoperations and shorter hospital stays compared to free flap reconstruction [9]. Local flaps present a dependable choice for addressing defects in the middle and distal regions of the lower extremities. This reconstruction method is suitable for small to medium-sized defects and can be safely utilized even in patients with comorbidities, such as diabetes mellitus and peripheral arterial occlusive disease, which can hinder wound healing [10]. Figure 1 illustrates a dorsalis pedis local flap of a male patient with a diabetic ulcer.

### Free flaps

In the lower extremity, the scarcity of soft tissues and compromised blood flow can pose challenges for reconstructive surgery, often necessitating microsurgical free flap reconstruction as the sole recourse for preserving the limb [2, 11]. Preserving the lower extremities enhances survival rates among multimorbid patients afflicted with nontraumatic defects [12]. Performing limb-sparing procedures alongside soft tissue reconstruction following oncological surgery should not be restricted solely to patients with curative intentions. Even patients in a palliative stage of the disease can experience pain relief and enhancements in quality of life through such surgical interventions [13]. An indicator for the likelihood of requiring secondary wound closure surgery is the presence of diabetes as a comorbidity [14].

The selection of the suitable flap considers factors such as the size of the defect, the patient’s overall health condition and the required tissue characteristics. Additionally, it is essential to confirm the presence of an appropriate connecting vessel in the vicinity of the defect. Particularly in extremities, angiography should be performed beforehand to assess the condition of the connecting vessels prior to surgery [15].

Drawing from Godina’s research, in cases of traumatic defects, early free tissue transfer has been linked to fewer complications compared to delayed reconstruction and is the optimal approach. The safe window for early soft tissue coverage can be extended to within 10 days of the injury. Various factors determine the time of reconstruction, such as the patient’s overall health and the contamination level of the defect [16].

The aim of this retrospective study was to assess the differences in reconstruction of the lower extremity by local flaps or free tissue transfer in traumatic and nontraumatic defects regarding limb preservation 12 months postoperatively. Postoperative morbidity and flap-associated complications were analyzed. 

## Material and methods

This retrospective investigation examined differences in lower extremity reconstruction using either local flaps or free tissue transfer for traumatic and nontraumatic defects, focusing on limb preservation and postoperative morbidity as well as flap-related complications, based on the chosen reconstructive treatment approach.

Approval for the study protocol was obtained from the Ethics Committee of the Medical University of Vienna (EK-number 1726/2019). Data for the study were extracted from patients’ medical records and operative notes.

Patients aged 18 years and above were included in this study, while pediatric patients were excluded. Patients with incomplete records or those who underwent extremity reconstructions at a different hospital were also excluded from the analysis.

A total of 187 patients with distal lower extremity defects underwent surgical reconstruction using either local or free flaps and were included in this retrospective study. Of the patients 104 received treatment involving free flap procedures, while 83 patients underwent local flap reconstruction.

The patient groups were divided by surgical reconstruction method. Patient demographics, the etiology and anatomical location of the defect requiring reconstruction and comorbidities were assessed. Postoperative follow-up times were analyzed. The main endpoint in both groups was limb preservation and complications during the follow-up period of 12 months.

To assess the outcome of the flap after surgery, flap-related complications were identified. Postoperative complications were categorized into minor and severe complications. Severe complications required surgical revision, whereas minor complications were resolved through conservative measures.

### Statistical analysis

Statistical analysis was conducted using SPSS software (version 24.0 for Windows, IBM SPSS Statistics, Inc, Chicago, IL, USA). Categorical data were presented as absolute frequencies and percentages, both for the entire study population and for each treatment group (local flaps and free flaps) separately. Bar charts were utilized to illustrate the findings. For metric variables, tables were used to display the mean, standard deviation, median, minimum and maximum values, along with the number of valid observations for each variable. Normality was assessed using the Shapiro–Wilk test prior to analysis. Parametric and nonparametric tests were used for group comparisons.

## Results

The 187 patients included in this study were categorized into 2 groups based on the surgical treatment. One group received treatment with a free tissue transfer (*n* = 104), while the other group underwent local flap surgery (*n* = 83).

Overall, 104 free tissue transfers were performed, involving 62 (59.6%) male and 42 (40.4%) female patients. The local flap group consisted of 84 patients, comprising 46 (54.7%) men and 38 (45.2%) women.

The average age of all patients was 52.27 years (SD ±17.68 years), ranging from 19–87 years. In the free tissue transfer group, the average age was 49.0 years (SD ±16.6 years), with a range of 19–87 years. The local flap group had an average age of 56.4 years (SD ±18.1 years; range: 20–87 years). A significant age difference was observed between the two groups (*p* = 0.004), as the average age of patients treated with free flap reconstruction was significantly younger.

The distribution of local flap types can be found in Table 1. The most commonly used local flaps were the gastrocnemius medialis flap (13.9%), the soleus flap (7.5%) and the gastrocnemius lateralis flap (5.9%).

**Table 1:** Local flap used to reconstruct lower extremity defects (*n* = 83)

Local flap type	*n*	Percentage
Gastrocnemius medialis	26	13.9
Soleus	14	7.5
Gastrocnemius lateralis	11	5.9
Plantaris medialis	7	3.7
Suralis	6	3.2
Random pattern local flap	5	2.7
Hemisoleus	4	2.1
Limberg flap	3	1.6
Dorsalis pedis	3	1.6
Extensor hallucis longus	2	1.1
Plantar	2	1.1

In the free tissue transfer group, the most performed flap was the latissimus dorsi (28.9%) followed by the gracilis flap (19.8%), shown in Table 2.

**Table 2:** Free flaps used for reconstruction of lower extremity defects (*n* = 104) *TRAM* transverse rectus abdominis muscle flap

Free flap type	*n*	Percentage
Latissimus dorsi	54	28.9
Gracilis	37	19.8
Fibula	9	4.8
Serratus anterior	2	1.1
TRAM	1	0.5
Random pattern flap	1	0.5

The most common etiologies of the lower extremity defects were trauma (29.3%), tumor genesis (25.0%) and postoperative defects (14.6%). This study included patients with defects in different anatomical regions of the distal lower extremity. Among these, the most common site was the knee (19%), followed by the middle third of the lower leg (17%) and the distal third of the lower leg (14%). Table 3 provides a detailed overview of the anatomical locations where free tissue transfers were performed.

**Table 3:** Anatomical location and performed free flap. (*n* = 104). *TRAM* transverse rectus abdominis muscle flap

Location	Free flap	*n*
Knee	Latissimus dorsi	16
Proximal 1/3 of the lower leg	Fibula	2
	Latissimus dorsi	2
	Gracilis	1
	Serratus anterior	1
Distal 1/3 of the lower leg	Fibula	3
	Latissimus dorsi	8
	Gracilis	10
Heel	Latissimus dorsi	4
	Gracilis	5
	Serratus anterior	1
	Random pattern flap	1
Lateral foot	Gracilis	6
	Latissimus dorsi	3
Achilles tendon	Latissimus dorsi	1
Lower 2/3 of the lower leg	Latissimus dorsi	1
	Gracilis	2
Lateral malleolus	Latissimus dorsi	2
	Gracilis	5
Dorsum of the foot	Fibula	4
	Latissimus dorsi	6
	Gracilis	6
	TRAM	1
Middle 1/3 of the lower leg	Latissimus dorsi	10
	Gracilis	1
Malleolus medialis	Latissimus dorsi	1
	Gracilis	1

Postoperative morbidity and flap-associated complications were analyzed by a reconstructive treatment concept. In the local flap group, the extremity could be preserved in 98.8%. In the group undergoing free tissue transfer, the rate of lower limb loss was 9.6%. Postoperatively, the overall survival rate of extremities was 97.2%. There was no statistically significant difference between the local and the free flap group.

Group comparisons showed no significant variation in flap loss rates based on the patients’ ages. Complications related to flaps in the local flap group requiring surgical revisions included 8 cases of complete flap loss and 3 hematoma evacuations. Partial flap necrosis occurred in 4 patients. The majority of patients (86.7%) experienced no severe complications.

In the free flap group, complications related to flaps included 19 cases of flap loss, 10 hematoma evacuations, 3 instances of venous thrombosis, and 3 instances of arterial thrombosis (21.9%).

The age of the patients did not significantly affect the lower leg amputation rate after 1 month or 12 months postoperative follow-up. Additionally, data on comorbidities were collected for patients in the local flap group. Of the patients 17 (20.5%) reported having comorbidities, such as peripheral artery occlusive disease (47.1%), diabetes (23.5%) and obesity (23.5%).

In the free flap group, 14 patients presented with comorbidities. The most common comorbidities were diabetes (64.3%), peripheral artery occlusive disease (21.4%), and obesity (14.3%). The Pearson χ^2^-test indicated no significant difference in the comorbidity rates between the two groups.

There was no significant difference in lower extremity reconstruction outcomes between local flaps and free flaps, whether for traumatic or nontraumatic defects, with respect to limb preservation at 12 months postoperatively. Additionally, morbidity and complications associated with the flaps were not significantly different between the two groups. Overall, our findings suggest that using either a free flap or a local flap yields similar outcomes in terms of limb preservation at 12 months postsurgery. Similarly, postoperative morbidity and complications were comparable across both groups.

## Discussion

This study provides a comprehensive analysis of reconstructive outcomes for lower extremity defects using local flaps and free tissue transfers. The findings highlight that both surgical approaches achieve comparable rates of limb preservation and postoperative morbidity at 12 months. While free tissue transfers were predominantly used for larger more distal defects, local flaps remained effective for smaller and proximal defects, reflecting the tailored application of each technique based on defect characteristics and patient factors. The literature indicates that similar causes are reported for defects in the lower extremities [17–20].

Flap selection for the distal lower extremity is determined by the size and anatomical location of the tissue defect. The choice of flap also considers donor site morbidity and aims to optimize the functionality of the lower extremity [18, 21, 22].

In many cases lower extremity defects go hand in hand with multiple comorbidities of the patients that can impact healing outcomes. Therefore, careful consideration of reconstructive options tailored to functional goals and risk factors is essential [23, 24].

The most frequently treated defect locations in this study were the knee region, the lateral malleolus and the proximal tibia.

The dimensions of the defect, vascular status of the lower extremity and patients’ comorbidities were described to determine treatment algorithms similar to those found in our study [22]. Consistent reconstructive results in larger defects can be achieved with latissimus and gracilis flaps as reported in previous studies [25]. One of the most common local flaps for the knee region and the proximal third of the lower leg was the gastrocnemius muscle flaps, which was consistent with the literature [26].

The progressive understanding of flap perfusion and design has elevated the reconstructive options in local and free flap applications and at the same time minimizing donor site morbidity [27, 28].

Free flap reconstruction was particularly advantageous for covering larger wounds in the distal lower extremity without additional trauma to local donor sites. It has become a pivotal step in the reconstructive ladder, with improved safety and efficacy supported by advancements in microsurgery [29, 30].

In this study the most commonly used free flaps were the latissimus dorsi and gracilis muscle flaps, recognized for their reliability in microsurgical practice and both often described as the ”workhorse“ of plastic and reconstructive surgery [31]. Free tissue transfer has demonstrated effectiveness in both traumatic and nontraumatic cases of lower extremity reconstruction, with flap selection influenced by patient-specific factors, timing of reconstruction, leg vascularity and defect characteristics [32, 33].

While both local and free flap reconstructions can contribute to extremity preservation, distal lower extremity cases are still associated with higher rates of postoperative complications [1, 34]. Postoperative complications are manageable with a high flap survival rate [6].

Limitations in this study are given through the retrospective character, the limited patient numbers and the limited types of flaps included in the analysis. Perforator flaps were not analyzed in this study due to the earlier period of some surgeries in our dataset, when the technique was less commonly employed.

Future studies should address the functional outcomes after lower extremity reconstruction, patient satisfaction and effects on quality of life. Furthermore, the postoperative setting should be analyzed in greater detail, like the self-efficacy of the patient.

Additionally, as these data were collected from a single institution with multiple reconstructive surgeons, variations in our patient population and surgical skills may limit the findings.

## Conclusion

This study provides valuable insights into the outcomes of free tissue transfers and local flap surgeries in a cohort of 187 patients. The findings highlight significant differences in the age distribution between the two groups, with younger patients predominantly undergoing free tissue transfers. Despite this, age did not significantly impact critical postoperative outcomes, including flap loss rates or limb preservation rates.

Overall, both surgical techniques demonstrated efficacy in reconstructive plastic surgery, with outcomes largely independent of patient age and comorbidity rates. These findings underline the importance of individualized treatment planning, taking patient-specific factors and the complexity of the surgical needs into consideration. Further longitudinal studies may help clarify the long-term implications of these surgical approaches and optimize patient outcomes.

## Data Availability

The underlying research data can be accessed by contacting the corresponding author at eva.placheta@meduniwien.ac.at
